# Genetic and Clinical Heterogeneity in Thirteen New Cases with Aceruloplasminemia. Atypical Anemia as a Clue for an Early Diagnosis

**DOI:** 10.3390/ijms21072374

**Published:** 2020-03-30

**Authors:** Marc Vila Cuenca, Giacomo Marchi, Anna Barqué, Clara Esteban-Jurado, Alessandro Marchetto, Alejandro Giorgetti, Viorica Chelban, Henry Houlden, Nicholas W Wood, Chiara Piubelli, Marina Dorigatti Borges, Dulcinéia Martins de Albuquerque, Kleber Yotsumoto Fertrin, Ester Jové-Buxeda, Jordi Sanchez-Delgado, Neus Baena-Díez, Birute Burnyte, Algirdas Utkus, Fabiana Busti, Gintaras Kaubrys, Eda Suku, Kamil Kowalczyk, Bartosz Karaszewski, John B. Porter, Sally Pollard, Perla Eleftheriou, Patricia Bignell, Domenico Girelli, Mayka Sanchez

**Affiliations:** 1 Iron Metabolism: Regulation and Diseases Group, Josep Carreras Leukaemia Research Institute (IJC), Campus Can Ruti, Badalona, 08916 Barcelona, Spain; m.vila.cuenca@gmail.com (M.V.C.); annabarque@gmail.com (A.B.); darth.clara@gmail.com (C.E.-J.); 2 EuroBloodNet Referral Center for Iron Disorders and Gruppo Interdisciplinare Malattie del Ferro, Internal Medicine Unit, Azienda Ospedaliera Universitaria Integrata di Verona, 37134 Verona, Italy; markallbutone@gmail.com (G.M.); fabiana.busti@gmail.com (F.B.); 3 Department of Biotechnology, University of Verona, 37134 Verona, Italy; alessandromarchetto@outlook.it (A.M.); alejandro.giorgetti@univr.it (A.G.); eda.suku@univr.it (E.S.); 4 National Hospital for Neurology and Neurosurgery, London WC1N 3BG, UK; v.chelban@ucl.ac.uk (V.C.); h.houlden@ucl.ac.uk (H.H.); n.wood@ucl.ac.uk (N.W.W.); 5 Department of Neuromuscular Diseases, Institute of Neurology, University College London, London WC1N 3BG, UK; 6 Department of Neurology and Neurosurgery, Institute of Emergency Medicine, Toma Ciorbă 1, Chisinau, MD-2052 Chisinau, Republic of Moldova; 7 Neurogenetics Laboratory, The National Hospital for Neurology and Neurosurgery, London WC1N 3BG, UK; 8 Centre for Tropical Diseases, Ospedale Sacro Cuore - Don Calabria, 37024 Negrar (VR), Italy; chiara.piubelli@univr.it; 9 Hematology and Hemotherapy Center—Hemocentro Campinas, University of Campinas—UNICAMP, Campinas 13083-878, Brazil; ma.borges@yahoo.com.br (M.D.B.); dulmal@unicamp.br (D.M.d.A.); kleber@uw.edu (K.Y.F.); 10 Division of Hematology, Department of Medicine, University of Washington, Seattle, WA 98195, USA; 11 Internal Medicine Department, Parc Tauli Hospital Universitari, Institut d’ Investigació i Innovació Parc Tauli I3PT, Universidad Autonoma de Barcelona, 08208 Sabadell, Spain; ejoveb@tauli.cat; 12 Hepatology Unit, Digestive Diseases Department, Parc Tauli Hospital Universitari. Institut d’ Investigació i Innovació Parc Tauli I3PT, Universidad Autonoma de Barcelona, 08208 Sabadell, Spain; jsanchezd@tauli.cat; 13 Centro de Investigación Biomedica y en red Enfermedades hepáticas y digestivas (CIBERehd), Instituto de Salud Carlos III, 28029 Madrid, Spain; 14 Genetic Department, Parc Tauli Hospital Universitari, Institut d’ Investigació i Innovació Parc Tauli I3PT, Universidad Autonoma de Barcelona, 08208 Sabadell, Spain; NBaena@tauli.cat; 15 Department of Human and Medical Genetics, Institute of Biomedical Sciences, Faculty of Medicine, Vilnius University, LT-08661 Vilnius, Lithuania; birute.burnyte@gmail.com (B.B.); algirdas.utkus@mf.vu.lt (A.U.); 16 Clinic of Neurology and Neurosurgery, Institute of Clinical Medicine, Faculty of Medicine, Vilnius University, 08661 Vilnius, Lithuania; gintaras.kaubrys@santa.lt; 17 Department of Adult Neurology, Medical University of Gdańsk, 80-210 Gdańsk, Poland; kkowalczyk@gumed.edu.pl (K.K.); bartkar@gumed.edu.pl (B.K.); 18 Joint Red Cell Unit, Haematology Department, University College London NHS Foundation Trust, Cancer Services, 250 Euston Road, London NW1 2PG, UK; j.porter@ucl.ac.uk (J.B.P.); perla.eleftheriou@nhs.net (P.E.); 19 Consultant Paediatrician, Bradford Royal Infirmary, Duckworthlane, Bradford BD9 6RJ, UK; sally.pollard1@nhs.net; 20 Oxford Regional Genetics Laboratory, Oxford University Hospitals NHS Foundation Trust, The Churchill Hospital, Oxford OX3 7LE, UK; patricia.bignell@ouh.nhs.uk; 21 Iron Metabolism: Regulation and Diseases Group, Department of Basic Sciences, Faculty of Medicine and Health Sciences, Universitat Internacional de Catalunya (UIC); Sant Cugat del Valles, 08017 Barcelona, Spain; 22 Program of Program of Predictive and Personalized Medicine of Cancer (PMPPC), Institut d ‘Investigació Germans Trias i Pujol (IGTP), Campus Can Ruti, Badalona, 08916 Barcelona, Spain; 23 BloodGenetics S.L., Esplugues de Llobregat, 08950 Barcelona, Spain

**Keywords:** aceruloplasminemia, ceruloplasmin, iron metabolism, neurodegenerative disease, anemia, ferritin

## Abstract

Aceruloplasminemia is a rare autosomal recessive genetic disease characterized by mild microcytic anemia, diabetes, retinopathy, liver disease, and progressive neurological symptoms due to iron accumulation in pancreas, retina, liver, and brain. The disease is caused by mutations in the Ceruloplasmin (*CP*) gene that produce a strong reduction or absence of ceruloplasmin ferroxidase activity, leading to an impairment of iron metabolism. Most patients described so far are from Japan. Prompt diagnosis and therapy are crucial to prevent neurological complications since, once established, they are usually irreversible. Here, we describe the largest series of non-Japanese patients with aceruloplasminemia published so far, including 13 individuals from 11 families carrying 13 mutations in the *CP* gene (7 missense, 3 frameshifts, and 3 splicing mutations), 10 of which are novel. All missense mutations were studied by computational modeling. Clinical manifestations were heterogeneous, but anemia, often but not necessarily microcytic, was frequently the earliest one. This study confirms the clinical and genetic heterogeneity of aceruloplasminemia, a disease expected to be increasingly diagnosed in the Next-Generation Sequencing (NGS) era. Unexplained anemia with low transferrin saturation and high ferritin levels without inflammation should prompt the suspicion of aceruloplasminemia, which can be easily confirmed by low serum ceruloplasmin levels. Collaborative joint efforts are needed to better understand the pathophysiology of this potentially disabling disease.

## 1. Introduction

Aceruloplasminemia (ACP) (OMIM#604290, ORPHA48818) is an adult-onset rare autosomal recessive disorder due to mutations in the *CP* gene (3q24-q25) encoding ceruloplasmin (CP), a copper-containing ferroxidase involved in maintaining iron homeostasis [1,2]. The pathological basis of ACP includes mild microcytic anemia and iron overload in several tissues, which can lead to diabetes mellitus, liver disease, progressive neurodegeneration, and retinopathy [1]. ACP was first described in 1987 in a 52-year-old Japanese female suffering from retinal degeneration, diabetes mellitus, and blepharospasm [3]. Usually, the onset of clinical manifestations is around the fourth or fifth decade of life, while biochemical signs of mild microcytic anemia with low transferrin saturation (TSAT) and paradoxically high ferritin may be evident since childhood [2]. Its prevalence is estimated at about one per two million offspring in Japanese non-consanguineous marriages, while epidemiologic data in the non-Japanese population are substantially missing [1].

Diagnosis is usually based on the evidence of very low or undetectable levels of serum CP and clinical, biochemical, or radiologic signs of iron overload in target organs. Current treatments are mainly based on iron-chelating agents, which are quite effective in reducing liver iron accumulation and may prevent further brain iron deposition but are poorly effective in patients with already established neurologic damage [4,5,6]. Administration of fresh-frozen human plasma [7], vitamin E, and oral administration of zinc [8] have also been reported. The administration of a CP enzyme replacement therapy showed promising results in CP-knockout mice [9].

**Figure 1:**
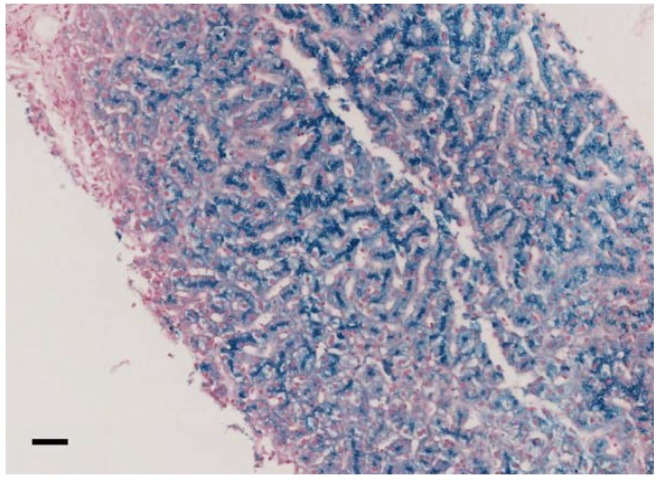
Liver histology of family 7 proband II.2, pearls stain (20×, scale bar = 100 μm). Biopsy analysis showed preserved liver architecture with marked iron deposition in hepatocytes, predominating at the biliary pole of the cells.

Understanding the pathophysiology of ACP is of interest also in other more common neurodegenerative disorders, such as Alzheimer’s and Parkinson’s diseases, characterized by an alteration of brain iron homeostasis [10]. CP is a single polypeptide chain of 1046 amino acids that can bind up to six atoms of copper. CP has six structural domains with a three-copper catalytic center, which is crucial for its oxidative function [11] and protein stability [12]. Two isoforms of CP are produced by alternative splicing in exons 19 and 20: a soluble form and a glycosylphosphatidylinositol (GPI)-anchored membrane form [13]. The soluble form is almost exclusively synthesized by hepatocytes, while the GPI-anchored membrane isoform is expressed by a number of cells, including brain astrocytes glial cells, hepatocytes, macrophages, pancreatic, and retinal epithelial cells [14]. This membrane isoform plays a key role in cellular iron egress cooperating with ferroportin, the ubiquitous and unique transmembrane protein able to export iron from the cells [15,16]. CP, with its copper-mediated oxidation of ferrous iron (Fe^2+^) to ferric iron (Fe^3+^), ensures the appropriate binding of extracellular iron to transferrin [1]. Since macrophages are key cells in iron recycling, a macrophage iron overload would be expected in ACP. Conversely, iron accumulation is usually observed in hepatocytes/liver (Figure 1) rather than in macrophages/spleen in affected patients, suggesting that additional pathophysiologic roles of CP are probably involved [17]. Specifically, it has been reported that GPI-anchored CP can also modulate the interaction between ferroportin and hepcidin [18,19,20], the master regulator of systemic iron homeostasis [21]. Furthermore, even if soluble CP is not able to pass the blood–brain barrier, it is also present in the brain through secretion by epithelial cells of the choroid plexus into the cerebrospinal fluid [22], and the role of this soluble form in the brain is still unclear. Overall, it emerges that CP is part of a complex regulatory system involved in maintaining iron balance, but further research is needed to better elucidate ACP pathophysiology, hopefully helping to develop more efficient treatments.

To address this need, in our international collaborative study, we describe the largest series of non-Japanese patients with ACP published so far, including 13 individuals from 11 families carrying 13 mutations in the *CP* gene, 10 of which are novel.

## 2. Patients and Methods

### 2.1. Patients

We got written informed consent from patients and relatives. Project: Uncovering new molecular and pathophysiological networks in iron metabolism (SAF2015-70412-R) approved on 10 July 2015 by the Ethics Research Committee from the Hospital Germans Trias i Pujol, Badalona, Spain. Protocol n.36792 from Project n.1460 was approved by the Ethics Research Committee from Hospital of Verona, Italy. Obtained informed written consent for molecular studies was obtained from all of the patients in accordance with the Declaration of Helsinki 1975, as revised in 2008 for The Diagnostic Lab Oxford Medical Genetics Laboratories from Oxford University Hospitals NHS Foundation Trust, United Kingdom. A detailed description of the biochemical, clinical, and genetic data of the 13 affected members in the 11 studied families with ACP is provided in the Supplementary Methods.

### 2.2. DNA Extraction, PCR Amplification, and DNA Sequencing

Genetic studies were performed with minor differences for all the pedigrees. Genomic DNA was extracted from peripheral blood using the FlexiGene DNA kit (Qiagen) or Wizard Genomic Purification kit (Promega, Madison, WI) or phenol-chloroform extraction (QIAsymphony DNA extraction kits, Qiagen) according to manufacturer’s instructions. 

Ceruloplasmin gene regions (exonic, intron-exon boundaries, and untranslated regions) were sequenced using the Sanger method or Next-Generation Sequencing (NGS) methods.

For Sanger methods, the *CP* gene was amplified using 50 ng of genomic DNA. Primer sequences and PCR conditions are available upon request. For patients in families 9 and 10, apart from sequencing the regions of the *CP* gene in which most mutations have been described [19], the whole CP pseudogene (*CPP*) was also sequenced. The resulting amplification products were verified on a 2% ethidium bromide agarose gel. The purified PCR products were sequenced using the conventional Sanger method. Sequencing results were analyzed using Mutation Surveyor software (SoftGenetics LLC, PA, USA) or Chromas software (Technelysium Pty Ltd., Australia).

For NGS methods, a patient in family 2 was analyzed using the targeted NGS gene panel (v14) for iron-related anemias that included the following 5 genes: *CP*, *TF*, *TMPRSS6*, *SLC11A2*, *STEAP3*. For patients in families 7 and 8, NGS sequencing was performed by targeted capture and sequencing of *CP*, along with the five canonical “hemochromatosis” genes (*HFE*, *HFE2*, *HAMP*, *TFR2*, and *SLC40A1*), as previously described [23]. Patients in families 4, 11, and 12 were analyzed using a targeted NGS gene panel including the following genes: *HFE*, *HJV*, *HAMP*, *TFR2*, *SLC40A1*, *ALAS2*, *CP*, *HEPH*, *TF*, *FTH1*, *FTL*, *SLC11A2*, *TMPRSS6*, *BMP4*, *BMP6*, and *SMAD4*.

Briefly, the capture of key genomic regions of interest was conducted starting from 225 ng of gDNA using a custom design HaloPlex^TM^ Target Enrichment 1–500 kb Kit (Agilent Technologies, Santa Clara, CA) or TruSeq^®^ Custom Amplicon version 1.5 kits (Illumina, CA) according to manufacturer instructions. Library quality was determined using the Agilent High Sensitivity DNA kit on the Agilent 2100 bioanalyzer or Agilent 4200 TapeStation System. Libraries were sequenced with the NextSeq 500 High Output Reagent Cartridge v2 (300 cycles) or MiSeq reagent kit v2 (300 cycles) (Illumina, San Diego, CA) on an Illumina NextSeq 500 or a MiSeq sequencer (Illumina, San Diego, CA), generating 150-bp paired-end reads. Samples were aligned with the reference human genome GRCh37/hg19 and data analysis was performed as previously described [24] or using the BasespaceTruSeq^®^ Amplicon App version 1.1 or own developed algorithms. Variant interpretation followed the American College of Medical Genetics and Genomics (ACMG) guidelines [25]. Mutations were confirmed by conventional Sanger sequencing.

Genetic variants are reported following official Human Genome Variation Sequence (HGVS) nomenclature and refer to NM_000096.3 for the *Homo sapiens CP* transcript variant, and NP_000087.1 for *Homo sapiens* ceruloplasmin precursor protein. Reported mutations in this study have been submitted to the Leiden Open Variation Database (http://www.lovd.nl) or to ClinVar (http://www.ncbi.nlm.nih.gov/clinvar) [26,27].

### 2.3. Bioinformatics and Computational Analysis

*In silico* predictions of missense variants, pathogenicity was performed using SIFT and Polyphen-2 bioinformatics tools [28,29].

Ceruloplasmin sequences from different species were retrieved from the PFAM database [30] and aligned using the MUSCLE program [31] for multiple alignments. Conservation analysis and alignment visualization were performed by Jalview software (version 2, www.jalview.org) [32].

The structural analysis of the missense variants was made based on the available human CP crystallographic structure [33]. Human Ceruloplasmin crystallographic structure was downloaded from the PDB server (PDB code: 4ENZ, crystallographic resolution: 2.6 Å; (http://www.rcsb.org/pdb/home/home.do). The Consurf server was used to map conservation features on the structure [34]. The prediction of the putative effects of the variants in the structure/function of the protein was performed by visual inspection using the Chimera program [35]. The wild-type residues and the modeled mutants were included in the IronGenes database, publicly accessible from: http://molsim.sci.univr.it/marchetto/php/gene_detail.php?geneId=CP#tabellaInit: from the first page, the results can be accessed graphically by selecting the ‘Variants Structures and Models’ button, and then directly exploring the different variants.

To analyze the plausible splicing effects of the CP c.1864+5G>A intronic variant, we used the bioinformatic NNSplice algorithm based on neural networks (http://www.fruitfly.org/seq_tools/splice.html [36]) and the Human Splicing Finder software [37] based on position-dependent logic.

## 3. Results

### 3.1. Clinical and Biochemical Profile

**Figure 2:**
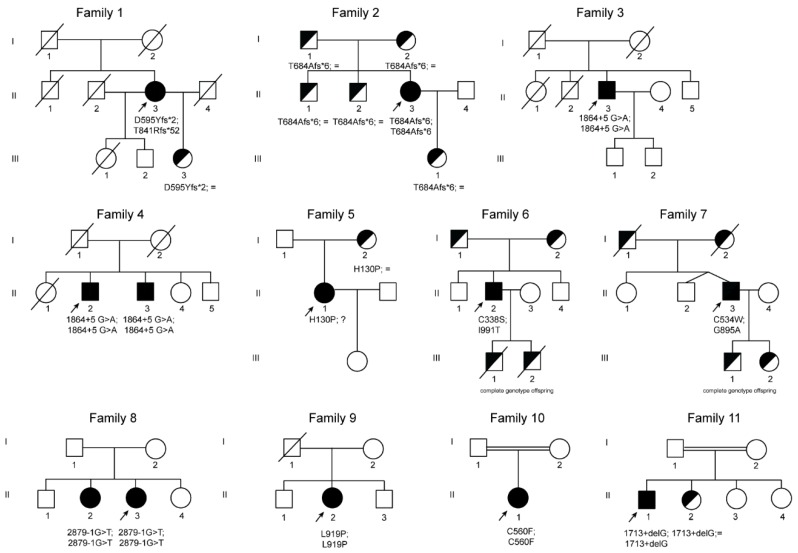
Pedigree trees from 11 studied families affected by ACP. Squares indicate males and circles females. Index cases are indicated with an arrow. Filled symbols indicate affected members, half-filled black symbols denote unaffected carriers, and barred symbols indicate deceased subjects. “?”, indicates individuals that were not possible to test because of refusal of testing or non-locatable person. Mutations are named according to the Human Genome Variation Sequence (HGVS) nomenclatures.

In this international collaborative study, we describe 11 new ACP families with 13 affected individuals (six men and seven women). Table 1 summarizes the biochemical, clinical, and genetic data of the patients. Pedigrees are shown in Figure 2. Family origins were spread worldwide, but none of the patients had Japanese ancestry (Table 1). Age at diagnosis ranged from 16 to 75 years (median 40 years), with an average diagnostic delay (i.e., time from clinical onset to the molecular diagnosis) of 10 years or more.

In our cohort, 11/13 (84.6%) patients had anemia (mild in 9/11), and 8/13 had microcytosis (61,5%). In all patients, serum ferritin values were high (geometrical mean 1409 ng/mL, CI95% 809.5-2452.5), and ceruloplasmin levels were very low or even undetectable. TSAT was decreased in 11/13 patients (84.6%), and serum iron was decreased in 10/13 patients (76.9%). Neurological symptoms were present in 75% of patients (data available from 12/13 patients) and ranged from severe complex neurological and psychiatric syndromes to mild cognitive dysfunctions. Brain iron overload at MRI was proven in ten (83.3%) of such patients. Eight out of nine patients (88.9%) had liver iron overload assessed by either liver biopsy (Figure 1) or MRI. Diabetes mellitus and retinopathy were present in 46.2% and 40% of cases, respectively. All patients are alive since our last contact with them.

### 3.2. Genetic Spectrum

Using a combination of Sanger sequencing and NGS, we identified 13 unique mutations, 10 of which are novel (see details in supplementary data).

Three novel frameshift mutations were detected in family 1 and family 2 (Figure 2, Table 1). Patient (II.3) from family 1 is compound heterozygous for two frameshift variants, the c.1783_1787delGATAA and c.2520_2523delAACA located in exon 10 and 14 of the *CP* gene, respectively. Both mutations lead to the creation of aberrant C-terminal CP protein ends and premature stop codons at position 596 and 892, respectively p.(Asp595Tyrfs*2 and Thr841Argfs*52). Proband (II.3) from family 2 has a novel homozygous deletion at position 2050 to 2051 of the *CP* gene leading to a premature stop codon at position 689 of ceruloplasmin protein p.(Thr684Alafs*6).

In families 3 and 4, sequencing analysis revealed in all probands and in one relative (brother of patient II.1 from family 4) a novel homozygous intronic mutation at position +5 downstream exon 10 of the *CP* gene (Figure 2 and Table 1). All individuals presenting this mutation are from India. The NNSplice algorithm program and the Human Splicing Finder splicing program predicted that the substitution of a G by an A at position +5 will abolish the wild-type donor splicing site of intron 10, interfering with *CP* splicing.

In family 5, a new missense mutation was detected in heterozygous state p.(His130Pro). His130 is engaged in a hydrogen bond with Tyr155, and its substitution with a proline causes the loss of this interaction (http://molsim.sci.univr.it/marchetto/php/gene_detail.php?geneId=CP#tabellaInit). Although this histidine is not well conserved in our multiple sequence alignment (Figure S1), in this position, there is always a polar or charged residue present, indicating that a substitution with a hydrophobic residue could cause protein misfolding. In this patient, we cannot exclude the existence of an additional mutation in non-studied regions (i.e., deep intronic or promoter regions). Interestingly, additional ACP cases with only one mutation and a second wild-type allele have also been previously described in the literature (see Table S1).

**Figure 3:**
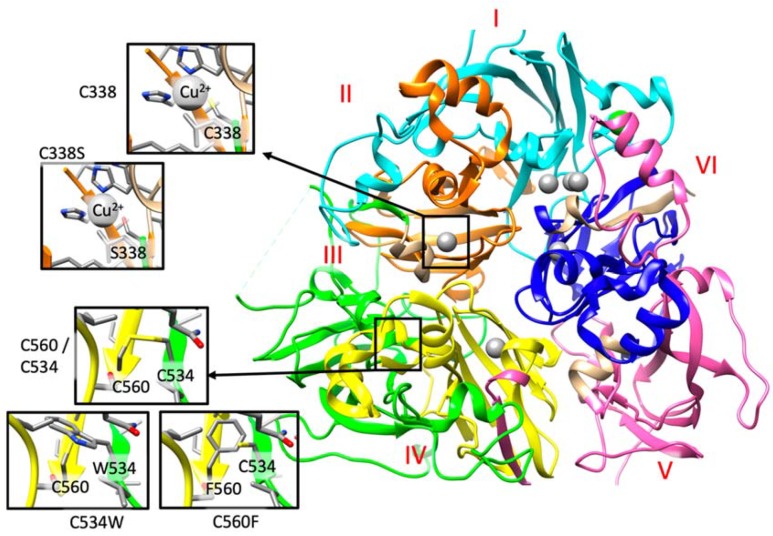
A. Detail of the structural analysis of selected missense mutations mapped on the structure of human ceruloplasmin (PDB code: 4ENZ). The reference and the mutated residue are indicated within the insights. The six Plastocyanin-like domains, described in the Uniprot Database (https://www.uniprot.org/uniprot/P00450) I to VI are colored in cyan, orange, green, yellow, pink, and blue, respectively. Copper domains are colored in grey. These three variants, as well as those detailed in the main text, can be retrieved from the IronGenes database (http://molsim.sci.univr.it/marchetto/php/gene_detail.php?geneId=CP#tabellaInit) developed at the University of Verona.

In family 6, the proband (II.2) is compound heterozygous for two *CP* missense mutations, p.(Cys338Ser) and p.(Ile991Thr). These two variants have been recently categorized as pathogenic mutations in ACP cases [38]. Indeed, Cys338 forms the binding cavity for one of the Cu^2+^ ions (Figure 3 and http://molsim.sci.univr.it/marchetto/php/gene_detail.php?geneId=CP#tabellaInit), and thus, the lack of a cysteine residue in that position could dramatically affect protein function.

In family 7, the patient is compound heterozygous for the missense mutation p.(Gly895Ala) (rs139633388, previously reported as p.(Gly876Ala) due to alternate nomenclature) and a novel missense variant, p.(Cys534Trp). The Gly895 is an evolutionary highly conserved (Figure S1) amino acid, and in silico modeling analysis indicated that its substitution by alanine could cause local perturbations due to size differences. Cys534 residue is involved in a disulfide bridge with Cys560 (Figure 2 and http://molsim.sci.univr.it/marchetto/php/gene_detail.php?geneId=CP#tabellaInit), and both cysteines are highly conserved (Figure S1); therefore, the p.(Cys534Trp) variant is predicted to have a high impact on protein stability.

In family 8, the proband and her elder sister carries a new splicing mutation (c.2879-1 G>T) in homozygosity in the donor site of intron 16 (variant rs386134141 and ClinVar ID 431112 reported by us). This mutation is predicted to lead to the loss of the splicing donor site located upstream of exon 17. A pathogenic mutation in the same position but with the change G>A (c. 2879-1 G>A, reported as nt2878-1G/A) was previously described as causative of ACP (see supplementary Table S1 and supplementary Reference 4).

In family 9, the proband is homozygous for a new missense mutation p.(Leu919Pro) in exon 16 (variant rs1135401784 and ClinVar ID 431113 reported by us). According to our computational model, Leu919 is involved in a network of hydrophobic interactions with Leu921 and Leu808 (http://molsim.sci.univr.it/marchetto/php/gene_detail.php?geneId=CP#tabellaInit) and is conserved in 86% of the sequences (Figure S1). The substitution by a proline would probably lead to perturbations in this network, altering the stability of the protein.

In family 10, the proband (II.1) is homozygous for a new missense mutation p.(Cys560Phe) in exon 9. Interestingly, Cys560 is 100% conserved (Figure S1) and is engaged in a disulfide bridge with Cys534; therefore, its substitution by Phe would have a high impact on the protein stability and/or structure (Figure 3 and http://molsim.sci.univr.it/marchetto/php/gene_detail.php?geneId=CP#tabellaInit). The effect, breaking of a disulfide bridge, is the same as the one encountered for the mutant Cys534 in family 7, pinpointing to a critical functional region of the protein.

In family 11, the proband (II.1) is homozygous for a new splicing mutation in intron 9, c.1713+1delG, which alters the donor splice site of exon 9, which is predicted to result in the skipping of exon 9. His cousin has the same phenotype and his sister is a carrier of this splicing mutation.

In Table S1, we provide an updated overview of all ACP cases reported in the literature and, if available, the corresponding *CP* mutations. Including our cohort, 107 patients with ACP from 83 families with a worldwide distribution were identified. Most reported cases are of Japanese origin, followed by Italian cases. In total, there are 71 different reported mutations causing ACP, distributed along the CP gene. All types of mutations have been reported (13 splicing mutations, 28 missense, 8 nonsense, 19 frameshift, and 2 big deletions), including 10 novel mutations that we reported in this study (3 splicing, 4 missense, and 3 frameshift mutations) (Figure S2). Most *CP* mutations are restricted to a particular family.

## 4. Discussion

**Figure 4:**
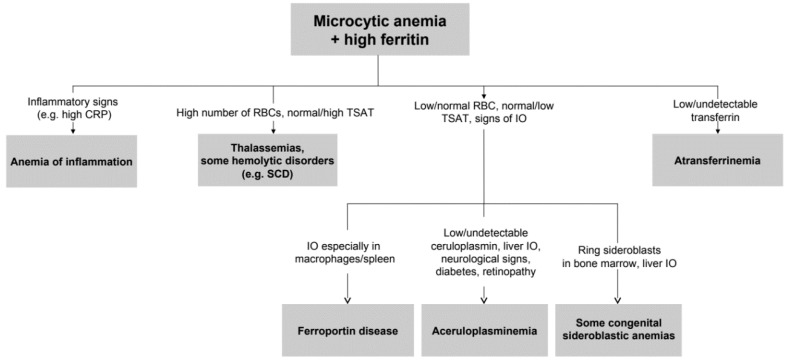
Simplified approach to microcytic anemia + high ferritin. From relatively simple laboratory tests also rare diseases can be suspected. Further investigations should include genetic testing, MRI for estimation of liver iron content, and eventually liver or bone marrow histopathology. IO: iron overload. SCD: sickle cell disease.

To the best of our knowledge, this study represents the largest series of non-Japanese ACP patients reported so far. The median age at diagnosis was 40 years, and the median diagnostic delay was about 10 years. Clinical and biochemical features of patients highlight substantial phenotypic heterogeneity, as also reported by a recent Italian case series [38], which contributes to the difficulties in the diagnosis of such a rare disease. The complete clinical triad of retinal degeneration, dementia, and diabetes, cited in a previous Japanese series [39], was present only in one 79-year-old patient (proband II.3 from family 1). Our data support that unexplained anemia, often but not necessarily microcytic, in association with low TSAT and paradoxically high ferritin are the best clues for an early diagnosis, which is crucial to prevent irreversible neurological manifestations. This type of anemia can be defined as “atypical microcytic anemia” to differentiate from other common causes of microcytosis, such as iron deficiency anemia or thalassemias [40,41,42]. With the exception of iron-refractory iron-deficiency anemia (IRIDA), atypical microcytic anemias are accompanied by progressive iron overload. In this regard, we propose a simplified algorithm for the diagnosis of ACP starting from microcytic anemia and hyperferritinemia in Figure 4.

In our series, neurological involvement and diabetes were present in 75% and 46.2% of patients, respectively, substantially in line with a previous case series [38,43,44]. Retinopathy was described in 40% of our patients, while it is reported in 76% of Japanese ACP patients [44]. Additional sporadic clinical findings in our series included hypothyroidism, amenorrhea, abdominal pain, and chronic diarrhea, but it was not possible to prove a causative role of ACP, which remains uncertain.

A genetic study of our patients also confirmed genetic heterogeneity. We detected 13 unique mutations, 10 of which are novel and distributed throughout the entire gene. Indeed, the aminoacidic changes affected all the six structural domains, with presumable different functional effects. The in-silico modeling provided in our work (and available at the link http://molsim.sci.univr.it/marchetto/php/gene_detail.php?geneId=CP#tabellaInit) is an attempt to further clarify the functional role of these mutations.

A description of new mutations, their functional study, and genotype–phenotype correlations are important for several reasons in ACP, including elucidate pathophysiology, understand why some patients develop brain iron accumulation and others do not, help in understanding brain iron metabolism also in other more common neurodegenerative disorders, and hopefully help in developing new and more efficient treatments.

## 5. Conclusions

Our study points out that ACP is distributed worldwide and is characterized by heterogeneous genotypes and phenotypes, requiring a high level of suspicion for diagnosis. Atypical anemia, often with microcytosis, low TSAT, and high ferritin, is the best clue for early pre-symptomatic diagnosis, which is crucial to avoid treatment delay and the onset of irreversible neurological damage. Collaborative joint efforts are needed among specialized laboratories and centers, as in our work, for a better definition of the molecular and clinical features of ACP, hopefully leading to new pathophysiological and therapeutical insights for this potentially incapacitating disease.

## Figures and Tables

**Table 1:** Biochemical, clinical, and genetic data of the 13 affected members in the 11 studied families with ACP.

Characteristic	Family 1	Family 2	Family 3	Family 4	Family 4	Family 5	Family 6	Family 7	Family 8	Family 8	Family 9	Family 10	Family 11	Normal Values
	II.3	II.3	II.3	II.2 (proband)	II.3	II.1	II.2	II.3	II.3 (proband)	II.2	II.2	II.1	II.1	
Country of Origin	Lithuania	Spain	India	India	India	Poland	Italy	Italy	Brazil	Brazil	Brazil	India	Pakistan	
Sex	F	F	M	M	M	F	M	M	F	F	F	F	M	
Age at Dx (years)	75	33	40	66	61	40	46	62	37	29	46	25	16	
Hb (g/dl)	11.1	10.9	12.1	11.6	10.7	11.1	12.5	12.2	11.7	12.3	9.4	9.2	13.4	12–16
MCV (fl)	82,0	85.1	77	66.8	81	88.0	84	70.6	75.2	71.4	64.5	71.5	69	79–99
Retyculocytes (%)	0.5	0.8	n/a	0.9	1.2	n/a	0.58	1	1.77	1.1	1.77	0.51	n.a.	1.1–2.7
RDW (%)	16.8	15.2	14.3	17.3	15.5	15.5	15.4	16.1	16	17.2	18	17.1	18	11.3–14.5
Serum iron (µg/dl)	82.8	15	28	19	39.1	81.0	33	215	23	23	22	9.5	33.5	37–170
Ferritin (ng/ml)	12159	355.4	1077	1112	3845	1143	2100	3650	791	732	1060	757	1065	10–290
Transferrin Saturation (%)	12.4	4.3	10	5	12	39	9	88	9.24	9.83	8.2	4	8.8	20–55
CP (mg/dl)	< 0.02	< 2.0	< 0.03	< 0.03	< 0.03	0.12	undetectable	0.12	11	9	< 2	n.a.	< 0.02	17–65
ALT (U/l)	55	12	32	24	36	19	37	113	26	179	37	24	87	14–36
AST (U/l)	43	17	n.a	n.a	24	16	20	80	12	82	29	n/a	n/a	8–40
Clue to ACP diagnosis	Low level of Cp. No Cu urine excretion Hepatocellular siderosis. Iron deposition in basal ganglia.	Low level of Cp. Low serum Cu and low urine Cu. Hepatic iron overload. Iron deposit in basal ganglia.	Iron deposition at brain MRI, Unexplained hyperferritinemia, low CP	Iron deposition at brain MRI, Unexplained hyperferritinemia, low CP	Iron deposition at brain MRI, Unexplained hyperferritinemia, low CP	Symptoms (including tremor) and very low CP	unexplained hyperferritinemia	overexpressed HFE Hemochromatosis, supposed additional non-HFE mutation(s) tested with NGS	Iron deposition at brain MRI	Familial investigation	Low CP in investigation of iron-refractory anaemia	Hair loss, mild executive dysfunction on formal neurocognitive assessment	low CP level	
Clinical presentation (symptoms and signs)	Moderate dementia with prevalent frontal features, cerebellar ataxia, oromandibular dystonia, torsion of the trunk, severe chorea-athetosis with choreiform movements. Mild type-2 DM. Retinal degeneration. Mild anaemi	Unspecific symptoms: Fatigue; abdominal discomfort and chronic anaemia. Liver ultrasonografy showed hepatic lesions that justified a MRI, that showed iron overload	Neurological symptoms: progressive cognitive decline, diabetes, mild bradykinesia with mild finger nose ataxia and dysdiadochokinesia .Unexplained anaemia	Neurological symptoms: progressive(over 5 years) cognitive decline, diabetes, tremor left hand Unexplained anaemia	Concentration and intellectual ability decline. Unexplained anaemia, DM.	Head and postural tremor of upper and lower limbs, slight dysmetria, ataxia, proximal weakness of lower extremities, horizontal nystagmus, and tunnel vision.	Liver iron overload and mild anaemia	Iron overload. Mild anaemia was consistent also with b-thalassemia trait	Choreiform movement disorder, mild anaemia, DM-2, asymptomatic retina pigmentation	Asymptomatic. Familial investigation	Mild anaemia, DM-2, asthenia, mild movement disorder	April 2015 acute psychotic episode with catatonia, hair loss, mild executive dysfunction on formal neurocognitive assessment	Concentration/memory lapses	
Anaemia	Yes	Yes	Yes	Yes	Yes	Yes	Yes	Yes	Yes	No	Yes	Yes	No	
Neurological symptoms	Yes	No	Yes	Yes	Yes	Yes	No	No	Yes	No	Yes	Yes	Yes	
Liver Iron overload	Yes	Yes	n.a.	Yes	Yes	No	Yes	n.a.	n.a.	Yes	n.a	Yes	Yes	
Diabetes	Yes	No	Yes	Yes	Yes	No	No	No	Yes	No	Yes	No	No	
Retinopathy	Yes	No	No	No	No	Not evaluated	Yes but not typical for aceruloplasminemia	No	Yes	Not evaluated	Not evaluated	No	Yes	
Brain MRI (sites of iron accumulation, in brief)	Iron overload in putamen	Iron overload in lenticular, dentate and thalamus	SWI increased susceptibility involving the cerebelum, basal ganglia, thalami, red and dentate nuclei.	MRI SWI with marked susceptibility predominantly involving the lateral putamen, red nucleus, striaum, thalamic, pulvinar, cerebellar dentate nucleus	Iron overload in lentiform caudate, dorsal lateral thalami and dentate nuclei	FLAIR and T2 hypointenseties in the putamen and substantia nigra	no iron overload	no iron overload	Iron overload in thalami, basal ganglia, and cerebellum	Iron overload in thalami, basal ganglia, red nuclei, dentate, cerebellum and brain cortex	Iron overload in thalami, basal ganglia, dentate, and cerebellum	Iron overload in choroid plexus, bilateral dentate nuclei thalamic and basal ganglia	n.a.	
Liver MRI or (biopsy)	hepatocellular siderosis grade III	HII = 9.58, severe iron overload	n.a.	Feriscan LIC: 9.4 mg/g dw	LIC by Ferriscan: 6.3 mg/g dw	Biopsy: small depositions of yellow-brown pigment (stain for ferrum - negative) - probably lipofuscin	LIC 340 µM/g = HII 7.9 (severe iron overload)	n.a.	not performed	Iron overload in liver and pancreas (qualitative)	not performed	LIC by Ferriscan 9.0 mg/g/dw	LIC by Ferriscan: 5.3 mg/g dw	HII < 1.9
Diagnostic delay	20 years	1.5 years	5 years	2 years	4 years	13 years	3 years	30 years	1 year	Not applicable	32 years	3 years	Not known	
Iron chelation or other therapy	No Iron chelation therapy.	Deferasirox from 10/2014 to 11/2015; Deferiprone from 11/2015; Vitamin E.	Started on FFP OctaplasLG every 2 weeks on diagnosis. Later added Deferiprone	Deferiprone started on diagnosis (25 mg/kg/d)	Deferiprone started 2011	Zinc	Deferasirox and desferoxamine both suspended for renal insufficiency, actually on deferiprone	Desferoxamine and “micro”-phlebotomies	Deferiprone	Desferoxamine, combination with deferiprone	Deferiprone	Deferiprone		
Other clinical data	Bilateral cataracts	Psoriasis	None	hypertension, previous CVA, low Vitamin B12, bilateral cataracts	2013: DM, 2011: hearing loss	Hypertension	3-4 alcoholic units/day, arterial hypertension, overweight, central serous chorioretinopathy	Beta-Thal trait; fully penetrant HFE-related HH (C282Y homozygous) in the 3rd decade of life	Hypercholesterolemia, macroalbuminuria	Hypothyroidism, lower limb venous thrombosis, migraine	Hypothyroidism, glaucoma, kidney stones, chronic diarrhea	Iatrogenic iron overload due to oral iron supplementation for microcytic anaemia, hypothyroid, amenorhoea (thought to be due to polycystic ovaries), microcytic anaemia, hypothyroid, borderline oral glucose tolerance test.	White nails, Acquired leukopenia, Bilateral lattice degeneration of fundi-risk of retinal detachment. Vit D depletion	
Genetics *CP* gene NM_000096.3; NP_000087.1	c.[1783_1787delGATAA(;)2520_2523delAACA] p.(Asp595Tyrfs*2;Thr841Argfs*52)	c.[2050_2051delAC]; [2050_2051delAC] p.(Thr684Alafs*6); (Thr684Alafs*6)	c.[1864+5G>A];[1864+5G>A]	c.[1864+5G>A];[1864+5G>A]	c.[1864+5G>A];[1864+5G>A]	c.[389A>C]; [=] p.(His130Pro); (=)	c.[1012T>A(;)2972T>C] p. (Cys338Ser);(Ile991Thr)	c.[2684G>C(;)1602T>G] p. (Gly895Ala);(Cys534Trp)	c.[2879-1G>T];[2879-1G>T]	c.[2879-1G>T];[2879-1G>T]	c.[2756T>C];[2756T>C] p.(Leu919Pro);(Leu919Pro)	c.[1679G>T];[1679G>T] p.(Cys560Phe);(Cys560Phe)	c.[1713+1delG];[1713+1delG]	

Reference values are indicated in the last column. Sex: M: male, F: female; ACP: Aceruloplasminemia; CP: ceruloplasmin; MRI: magnetic resonance imaging; SWI Susceptibility weighted imaging; HII hypoxic-ischemic injury; LIC: liver iron content; Hb: Hemoglobin; MCV: mean corpuscular volume; RDW: red cell distribution width; DM-2: Diabetes mellitus type 2; NGS: next-generation sequencing; n.a.: not available.

## Supplementary Materials

Supplementary materials can be found at https://www.mdpi.com/1422-0067/21/7/2374/s1.
