# Supplementary material for: Genetic and Clinical Heterogeneity in Thirteen New Cases with Aceruloplasminemia. Atypical Anemia as a Clue for an Early Diagnosis
Source: Int J Mol Sci. 2020 Mar 30;21(7):2374. doi: 10.3390/ijms21072374 (PMC7178074; doi:10.3390/ijms21072374)
Supplement: Supplementary file 1 [file ijms-21-02374-s001.pdf]

Genetic and Clinical Heterogeneity in Thirteen New Cases with Aceruloplasminemia. Atypical Anemia as a Clue for an Early Diagnosis

Table S1. Table summarizing all aceruloplasminemia cases and their mutations described up to now in the literature (including the cases reported in this publication).

Nº family	Nº patients	Country	Mutation 1 cDNA and (Protein)	Mutation 1 CADD PHRED Score	Mutation 2 cDNA and (Protein)	Mutation 2 CADD PHRED Score	Ref.
1	2	Japan	c.82A>T p.(Ile28Phe)<br>reported as p.(Ile9Phe)	26.1	WT	-	[1]
1	1	Belgium	c.146+1G>A	33	c.2603delG<br>p.(Gly868Glufs*26)<br>Reported as 1 bp del at ntd 2602 in exon 15	n/a	[2]
1	1	Armenia	c.229G>C p.(Asp77His)<br>Reported as p.(Asp58His)	25.4	c.2131C>A p.(Gln711Lys)<br>Reported asp.(Gln692Lys)	20.6	[3]
n/a	n/a	n/a	c.229G>C p.(Asp77His)<br>Reported as p.(Asp58His)	25.4	n/a	-	[4]
n/a	n/a	n/a	c.395-1G>A	34	n/a	-	[4]
1	1	Italy	c.493C>G p.(Gln165Glu)<br>Reported as p.(Gln146Glu)	16.08	c.2975dupA<br>p.(Asp992Glufs*11)<br>Reported as A ins 2917	n/a	[5]
1	1	Japan	c.583G>A p.(Gly195Arg)<br>Reported as p.(Gly176Arg)	29.7	WT	-	[6]
1	1	Japan	c.587C>G p.(Pro196Arg)<br>Reported as p.(Pro177Arg)	27.4	WT	-	[6-8]
n/a	n/a	n/a	c.548T>C p.(Ile183Thr)<br>Reported as p.(Ile163Thr)	24.5	n/a	-	[9]
1	1	Japan	c.587C>G p.(Pro196Arg)<br>Reported as p.(Pro177Arg)	27.4	n/a	-	[8]
1	1	Japan	c.606dupA<br>p.(Asp203Argfs*5)<br>Reported as nt.607 insA	n/a	n/a	-	[10]
1	2	Japan	c.607+1G>A	34	n/a	-	[11]
n/a	n/a	n/a	c.643C>T p.(Arg215Ter)<br>Reported as p.(Arg196Ter)	35	n/a	-	[4]
1	1	Italy	c.650T>C p.(Phe217Ser)	29.6	c.650T>C p.(Phe217Ser)	29.6	[12, 13]
1	2	China	c.848G>C p.(Trp283Ser)	33	c.848G>C p.(Trp283Ser)	33	[14]
1	1	Italy	c.848G>A p.(Trp283Ter)	40	c.2689_2690delCT<br>p.(Leu897Aspfs*27)	n/a	[15]
1	1	Italy	c.1012T>A p.(Cys338Ser)	27.9	c.1012T>A p.(Cys338Ser)	27.9	[13]
3	1	Spain	c.1049C>A p.(Ala350Asp)	28.6	c.1049C>A p.(Ala350Asp)	28.6	[16]
n/a	n/a	n/a	c.1123T>C p.(Tyr375His)	28.7	n/a	-	[4]
1	2	Italy	c.1208+1G>A	34	c.1208+1G>A	34	[13]
1	1	Japan	c.1209-2A>G	28.6	c.1209-2A>G	28.6	[17, 18]
1	2	India	c.1209_1210dupTG<br>p.(Asp404Valfs*61)<br>Reported as c.1211_1212dupTG	n/a	c.1209_1210dupTG<br>p.(Asp404Valfs*61)<br>Reported as c.1211_1212dupTG	n/a	[19]
1	2	Italy	c.1257_1258delTT<br>p.(Tyr420Ter)<br>Reported as p.(Tyr401X)	n/a	c.1257_1258delTT<br>p.(Tyr420Ter)<br>Reported as p.(Tyr401X)	n/a	[20]

1	3	Japan	c.1282_1286dupTACAC p.(Asp430Thrfs*36) Reported as 5bp ins at aa 410	n/a	c.1282_1286dupTACAC p.(Asp430Thrfs*36) Reported as 5bp ins at aa 410	n/a	[21, 22]
1	1	Turkey	c.1306C>T p.(Arg436Ter)	35	c.1306C>T p.(Arg436Ter)	35	[23]
n/a	n/a	n/a	c.1652C>T p.(Thr551Ile)	26.8	n/a	-	[9]
1	1	Japan	c.1864+1G>C	34	c.1864+1G>C	34	[24]
n/a	n/a	n/a	c.1865-1G>A	34	n/a	-	[4]
1	1	Italy	c.1865-292_2077+351del p.(Ser622_Gly693delinsTrp)	n/a	c.1865-292_2077+351del p.(Ser622_Gly693delinsTrp)	n/a	[13, 25]
1	1	Japan	c.1874G>A p.(Gly625Glu)	32	WT	-	[26]
1	1	France	c.1916_1918delGAGinsA p.(Gly639Glufs*13)	n/a	c.2139T>A p.(Tyr713Ter) Reported as p.(Tyr694X)	36	[27]
n/a	n/a	n/a	c.1918delG p.(Asp640Ilefs*70)	n/a	n/a	-	[4]
1	1	Italy	c.1948G>A p.(Gly650Arg) Reported as p.(Gly631Arg)	32	c.1948G>A p.(Gly650Arg) Reported as p.(Gly631Arg)	32	[28]
1	1	The Netherlands	c.1948G>A p.(Gly650Arg)	32	c.1948G>A p.(Gly650Arg)	32	[29]
1	4	The Netherlands	c.1948G>A p.(Gly650Arg) reported as p.(Gly631Arg)	32	c.1948G>A p.(Gly650Arg) reported as p.(Gly631Arg)	32	[30-32]
n/a	n/a	n/a	c.2066delC p.(Pro689Leufs*21) Reported as nt 2065 C del	n/a	n/a	-	[4]
1	1	Japan	c.2068delG p.(Asp690Thrfs*20)	n/a	c.2068delG p.(Asp690Thrfs*20)	n/a	[33]
1	1	Italy	c.2078-74_2241del	n/a	c.2078-74_2241del	n/a	[13, 34]
1	3	Germany	c.2122G>A p.(Gly708Ser)	27.9	c.2122G>A p.(Gly708Ser)	27.9	[35]
n/a	n/a	n/a	c.2131C>A p.(Gln711Lys) Reported as p.(Gln692Lys)	20.6	n/a	-	[4]
1	2	Germany	c.2158C>T p.(Arg720Trp)	14.56	WT	-	[36]
1	1	Italy	c.2158C>T p.(Arg720Trp)	14.56	WT	-	[37]
1	1	Japan	c.2185delC p.(Leu729Trpfs*40)	n/a	c.2185delC p.(Leu729Trpfs*40)	n/a	[38]
1	2	Ireland	c.2389delG p.(Glu797Argfs*12)	n/a	c.2389delG p.(Glu797Argfs*12)	n/a	[39]
n/a	n/a	Japan	c.2482delG p.(Ala828Profs*66)	n/a	n/a	-	[40]
1	1	Germany	c.2511dupT p.(Gly838Trpfs*16) Reported as c.2510 insT	n/a	c.2511dupT p.(Gly838Trpfs*16) Reported as c.2510insT	n/a	[41]
1	3	Austria	c.2554+1G>T	15.95	c.2554+1G>T	15.95	[42]
1	1	Japan	c.2603delG p.(Gly868Glufs*26) Reported as nt 2062 1bp del	n/a	c.2603delG p.(Gly868Glufs*26) Reported as nt 2062 1bp del	n/a	[43]
1	1	Japan	c.2603delG p.(Gly868Glufs*26) Reported as nt2602 delG	n/a	c.2603delG p.(Gly868Glufs*26) Reported as nt2602 delG	n/a	[44]
1	2	Japan	c.2630G>A p.(Trp877Ter) Reported as p.(Trp858Ter)	39	c.2630G>A p.(Trp877Ter) Reported as p.(Trp858Ter)	39	[45]
1	1	Japan	c.2630G>A p.(Trp877Ter) Reported as p.(Trp858Ter)	39	WT	-	[46, 47]
1	1	Japan	c.2630G>A p.(Trp877Ter) Reported as p.(Trp858Ter)	39	c.2630G>A p.(Trp877Ter) Reported as p.(Trp858Ter)	39	[48]
1	1	Japan	c.2630G>A p.(Trp858Ter)	39	WT	-	(46)

			Wrongly Reported as c.2360G>A				
n/a	n/a	n/a	c.2675G>A p.(Gly892Glu) Reported as p.(Gly873Glu)	27	n/a	-	[9]
n/a	n/a	Japan	c.2684G>C p.(Gly895Ala) Reported as p.(Gly876Ala)	26.7	n/a	-	[4]
n/a	n/a	n/a	c.2689_2690delCT p.(Leu897Aspfs*27)	n/a	n/a	-	[9]
1	1	Japan	c.2701C>T p.(Arg901Ter)	41	c.2991T>G p.(His997Gln)	23.3	[49]
n/a	n/a	n/a	c.2879-1G>A Reported as nt2878-1 G/A	33	n/a	-	[4]
n/a	n/a	n/a	c.2953A>G p.(Met985Val) Reported as p.(Met966Val)	18.49	n/a	-	[4]
1	1	Japan	c.2962G>A p.(Gly988Ser)	32	c.2962G>A p.(Gly988Ser)	32	[50]
1	1	Italy	c. 2972T>C p.(Ile991Thr)	23.2	c. 2972T>C p.(Ile991Thr)	23.2	[13]
1	4	Japan	c.3019-1G>A consequence c.3019_3023delCACAG p.(His1007Glyfs*4)	32	c.3019-1G>A consequence c.3019_3023delCACAG p.(His1007Glyfs*4)	32	[51, 52]
1	1	Japan	c.3107G>A p.(Trp1036Ter) Reported as p.(Trp1017X)	42	c.3107G>A p.(Trp1036Ter) Reported as p.(Trp1017X)	42	[53]
1	2	Brasil	n/a	-	n/a	-	[54]
1	3	France	n/a	-	n/a	-	[55]
1	1	USA	n/a	-	n/a	-	[56]
1	1	USA	n/a	-	n/a	-	[57]
1	n/a	USA	*c.1632del*		*c.391G > A*		[58]
1	n/a	Japan	c.1286_1290insTATAC		c.2185delC		[59]
1	n/a	The Netherlands	c.1192-1196del, p.Leu398Serfs		c.1192-1196del, p.Leu398Serfs		[60]
1 (F1)	1	Lithuania	c.1783_1787delGATAA p.(Asp595Tyrfs*2)	n/a	c.2520_2523delAACA p.(Thr841Argfs*52)	n/a	This study
1 (F2)	1	Spain	c.2050_2051delAC p.(Thr684Alafs*6)	n/a	c.2050_2051delAC p.(Thr684Alafs*6)	n/a	This study
1 (F3)	1	India	c.1864+5G>A	22.6	c.1864+5G>A	22.6	This study
1 (F4)	2	Indian	c.1864+5G>A	22.6	c.1864+5G>A	22.6	This study
1 (F5)	1	Poland	c.389A>C p.(His130Pro)	23.3	WT	-	This study
1 (F6)	1	Italy	c.1012T>A p.(Cys338Ser) [(a)]	27.9	c. 2972T>C p.(Ile991Thr) [(b)]	23.2	This study
1 (F7)	1	Italy	c.2684G>C p.(Gly895Ala) [(c)]	26.7	c.1602 T>G p.(Cys534Trp)	24.3	This study
1 (F8)	2	Brazil	c.2879-1G>T	33	c.2879-1G>T	33	This study
1(F9)	1	Brazil	c.2756T>C (p.(Leu919Pro)	25.9	c.2756T>C p.(Leu919Pro)	25.9	This study
1(F10)	1	India	c.1679G>T p.(Cys560Phe)	28.9	c.1679G>T p.(Cys560Phe)	28.9	This study

1(F11)	1	Pakistan	c.1713+1delG	n/a	c.1713+1delG	n/a	This study

The table shows for each mutation, the conventional nomenclature according to HGVS (corresponding to NCBI: NM_000096.3 and NP_000087.1 reference sequences), the original reported nomenclature, the number of families and patients described, the patients' ancestry and the corresponding published report and the CADD PHRED Score taken from https://cadd.gs.washington.edu/. (a) Mutation also reported in reference 56 in a different patient, (b) Mutation also reported in reference 56 in a different patient, (c) Mutation also reported in reference 4 in a different patient.

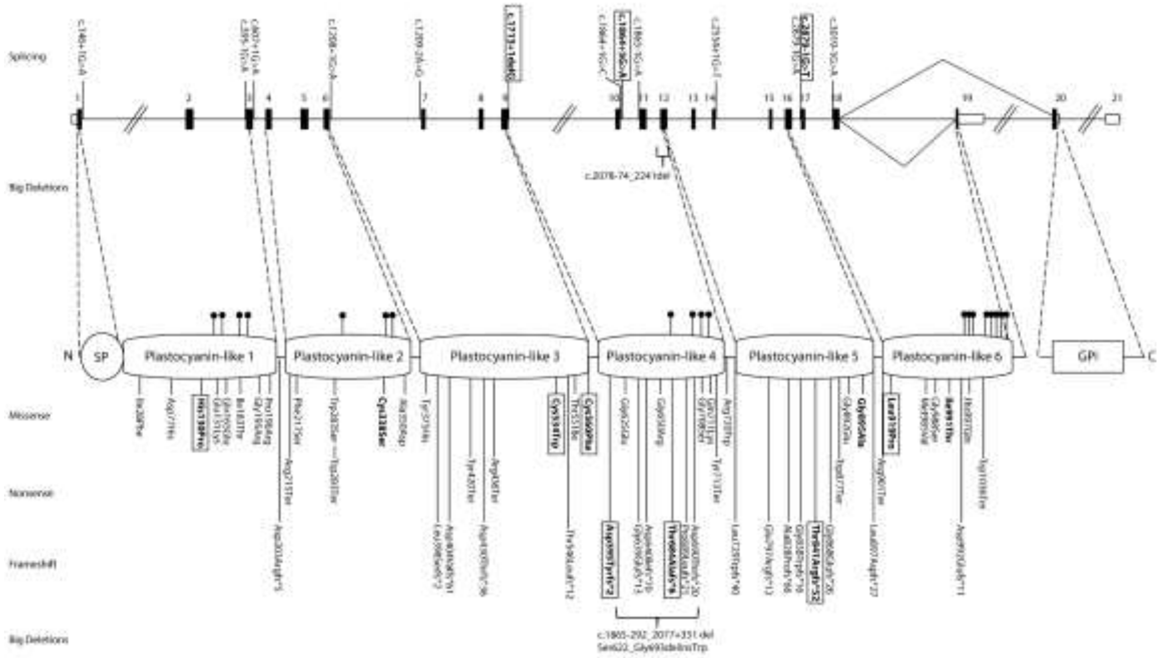

Figure S1. Schematic localization of *CP* mutations reported in literature and in our cohort. Mutations described in this work are in bold and novel mutations are boxed. Domains: SP, signal peptide, Cu-OXIDASE, copper oxidase; GPI, glycosylphosphatidylinositol anchor. Black circles represent aminoacids that are involved in binding of copper atoms according to UniProt (Accession number: P00450, http://www.uniprot.org/). Mutations are classified as nonsense, frameshift, missense and splicing. We report here the CP protein changes using the three-letter amino acid c

	H130	C338	C534	C560	G895	L919	I991
G	0,006667	0	0	0	0,966667	0	0
A	0,003333	0	0	0	0	0	0,006667
V	0,006667	0	0	0	0	0	0,413333
L	0	0	0	0	0	0,86	0
I	0	0	0	0	0	0	0,4
M	0	0	0	0	0	0,02	0,006667
F	0	0	0	0	0	0	0,073333
W	0	0	0	0	0	0	0
P	0	0	0	0	0	0	0
S	0,38	0	0	0	0	0	0,006667
T	0,013333	0	0	0	0	0	0,013333
C	0	0,973333	1	1	0	0	0
Y	0					0,006667	0,013333
N	0,266667	0	0	0	0	0	0
Q	0					0,006667	0,006667
D	0	0	0	0	0	0	0
E	0	0	0	0	0	0	0
K	0,006667	0	0	0	0	0	0,006667
R	0	0	0	0	0	0	0
H	0,153333	0	0	0	0	0,066667	0

Figure 2. Conservation analysis for aminoacidic residues involved in missense mutations, detected in our study (reported in header row). Numbers are calculated as x/100, where x indicates the occurrence of the corresponding amino acid (reported in the first column) out of 100 analysed sequences. Highly conserved residues (> 85%) are highlighted in red. Conservation analysis was performed using Jalview software (59).

Detailed supplementary patients and methods

Material and Methods

PATIENTS

Family 1

Proband II.3 from family 1 was a 79-year-old woman of Lithuanian origin presenting a history of neurological symptoms and retinal degeneration. She developed depression and mild cognitive impairment at the age of 54. At the age of 72, she was found to have moderate dementia, with more pronounced frontal features, but also signs of parietal and temporal involvement. Motor and cerebellar symptoms appeared the year after, with gait and cerebellar ataxia that resulted in frequent falls and traumas, and intention tremor. She also developed oromandibular dystonia and torsion of the trunk, severe chorea-athetosis with prevalent choreiform movements. Brain MRI showed moderate brain atrophy, and signs of iron deposition in the putamen, the caudate nucleus,

the posterior thalamus, and the dentate nucleus of the cerebellum. Biochemical analyses revealed mild anemia (Hb 11.0 g/dl), markedly elevated ferritin levels (12,159 ng/ml) and undetectable serum ceruloplasmin. Other biochemical parameters are reported in Table 1. A mild non-insulin dependent diabetes mellitus was also detected. Liver biopsy showed hepatocellular siderosis (grade III).

Family 2

Proband II.3 from family 2 was a 35-year-old female from Spain who referred because of worsening asthenia, weight loss, nausea without vomiting, and abdominal pain lasting for several months (Figure 1 and Table 1). The patient has a 20-year clinical history of mild microcytic anemia intermittently treated with oral iron. Laboratory analyses revealed low Hb (10.9 g/dl), low serum iron (15 μg/dl) and copper (12 μg/dl), elevated ferritin levels (355.4 ng/ml), decreased TSAT (8.5%), and undetectable ceruloplasmin, as well as anisopoikilocytosis. Brain MRI showed iron overload in lenticular and dentate nucleus, as well as in the thalamus. Liver MRI revealed severe iron accumulation confirmed by biopsy displaying a hepatic iron index of 9.58 (HII >1.9 is typically considered the hallmark of genetically determined iron overload (60)). No further causes of iron overload were apparent.

Family 3

The proband in family 3 is a 70-year-old male who presented at the age of 40 affected by diabetes mellitus, which did not respond to oral medication and required treatment with insulin with a very difficult glycaemic control. Several years later, routine blood tests showed apparently unexplained high ferritin levels (1,077 ng/ml) with reduced TSAT (10%) and serum iron (28 μg/dl). Complete Blood Count (CBC) showed mild microcytic anemia (Hb 12.1 g/dl, MCV 77.6 fl). Subsequent investigations showed a markedly low serum copper (4 μg/dl), and undetectable ceruloplasmin. He also presented a slowly progressive cognitive impairment, with loss of short-term memory. Neurological examination revealed a general slowness, mild bradykinesia with finger nose ataxia and dysdiadochokinesia. MRI showed iron deposition in basal ganglia, red and dentate nuclei.

Family 4

Two siblings from a non- consanguineous family of North Indian origin presented with a very similar phenotype. They also have another sister who died in her fifties from unspecified liver disease, as well as several unaffected siblings (Figure 1). The proband II.2 was a 71-year-old male. He had a nine-year history of progressive diabetes mellitus that was later associated with short-term memory problems, upper limbs postural tremor and

mild ataxia with bilateral cataracts. The MRI shows abnormal iron deposition in the cerebellum, external capsule, and substantia nigra bilaterally. The younger sibling presented with anemia-related fatigue, diabetes mellitus and a normal neurological examination with only minimal iron deposition seen within the lentiform, caudate, dorsal lateral thalami and dentate nuclei on MRI. Both siblings had undetectable ceruloplasmin levels with hyperferritinemia (ferritin levels >1,000 ng/ml in both cases) and mild microcytic anemia with low transferrin saturation (Table 1).

Family 5

Proband II.1 from family 5 was a 41-year-old Polish female presenting with a 10-year history of tremor (Figure 1 and Table 1). Neurological examination revealed head and postural tremor of upper and lower limbs, mild dysmetria, ataxia, proximal weakness of lower extremities, horizontal nystagmus, and tunnel vision due to retinal degeneration. None of her relatives has developed similar symptoms or was diagnosed with a neurological disorder. Three years after, mild anemia (Hb 11.1 g/dl) was observed, while CBC at presentation was reported normal. Brain MRI showed iron deposition in the putamen and substantia nigra. Differential diagnostic testing revealed very low serum ceruloplasmin (0.12 mg/dl) and copper (37 μg/dl) concentrations.

Family 6

Proband II.2 from family 6 was a 46-year-old Italian-male who was referred to a specialized iron clinic because of unexplained hyperferritinemia (2,100 ng/ml), detected after an oral iron supplementation (Figure 1 and Table 1). Indeed, two months earlier, the patient's general practitioner had prescribed ferrous sulphate 105 mg/day because of mild anemia found at routine CBC examination. The patient reported that a mild anemia was long detected since he was a child. His clinical history revealed an alcohol intake of 3-4 units/day, features of metabolic syndrome (arterial hypertension, overweight) possibly suggesting dysmetabolic hyperferritinemia, gastroesophageal reflux disease, and central serous chorioretinopathy, noticed 3 years before. He had never received blood transfusions. He was unaware of any relative affected by iron overload disorders; his mother was reported to be affected by iron deficiency anemia during two pregnancies, when she was supplied with iron with apparent benefit. He had two kids, both died early because of medulloblastoma. Clinical evaluation showed mild hepatomegaly, while neurological examination was normal. Further blood analyses showed low TSAT (9%), mild anemia (Hb 12.5 g/dl), MCV and MCH at lower limits (84 fl and 36 pg, respectively), low reticulocyte count (0.58 %). Transaminases and C-Reactive Protein were normal, while additional features of the metabolic

syndrome were evident (impaired fasting glucose, low HDL-cholesterol levels, and high levels of uric acid). Liver iron overload was confirmed by MRI (Liver Iron Content – LIC of 340 +/- 50 μM/g), as well as by liver biopsy (preserved liver architecture, 3+ iron deposits in hepatocytes at Pearl's staining). A diagnostic procedure included ceruloplasmin assay, showing extremely reduced levels (Table 1).

Family 7

Proband II.3 from family 7 was a 62-year-old Italian–male, who was referred to a tertiary iron clinic for consultation. He had previously diagnosed elsewhere with severe "fully penetrant" HFE-related hemochromatosis (HH), but some doubts arose. The patient, who was also affected by mild anemia due to ß-thalassemia trait, had a 30 years history of iron chelation therapy (Figure 1 and Table 1). Marked hyperferritinemia (3,650 ng/ml), with high TSAT (88%) was detected for the first time at the age of 30 years, during examination prior to a possible enrolment as blood donor (he was unaware of being thalassemic). Serum CP was undetectable. Liver transaminases were increased (AST 80 U/l, ALT 113 U/l), and a liver biopsy confirmed marked liver iron deposition consistent with the diagnosis of hemochromatosis (Figure 3). He was treated with phlebotomies along with iron chelation therapy. Some years after, when molecular testing for hemochromatosis became available, homozygosity for the p.Cys282Tyr mutation on the HFE gene was detected. At the recent re-evaluation the patient was still on low-dose iron chelation therapy, and asymptomatic. He was also affected by arterial hypertension and mitral valve prolapse. The clinical evaluation revealed mild hepatomegaly and splenomegaly and mild mitral regurgitation. The neurological examination was normal. Blood analyses showed mild hyperferritinemia (320 ng/ml), high TSAT (87%), and mild microcytic hypochromic anemia (Hb 12.2 g/dl, MCV 66 fl, MCH 21 pg). Liver function tests were normal. The earlyonset (third decade) HFE-related hemochromatosis suggested the possible presence of additional mutations, thus a recently validated NGS-based second level genetic test for iron genes (62) was performed. Indeed, in addition to homozygosity for the p.Cys282Tyr mutation in *HFE* gene and to heterozygosity for the ß-globin (*HBB*) mutation NM_000518.4:c.93-21G>A (rs35004220), a compound heterozygosity for two mutations in the *CP* gene was found (see main manuscript).

Family 8

Proband II.3 form family 8 was a 29-year-old female from Brazil who was referred because of the detection of brain iron overload upon MRI during investigations for migraines and mild movement disorder. MRI showed

iron overload in thalami, basal ganglia, red nuclei, dentate, cerebellum and brain cortex (Figure 1 and Table 1). Laboratory analyses showed microcytosis (MCV 71.4 fl) with borderline Hb levels (12.3 g/dl), low serum iron (23 μg/dl) and TSAT (9.8%), hyperferritinemia (732 ng/ml), and low ceruloplasmin levels (9 mg/dl). Her elder sister was then investigated, and she also showed mild microcytic anemia (Hb 11.7 g/dl, MCV 75.2 fl), with elevated ferritin (791 ng/ml), low TSAT (9.2%), and low ceruloplasmin (11 mg/dl). Remarkably, she had been diagnosed with diabetes mellitus five years earlier. After the diagnosis of aceruloplasminemia, she underwent a brain MRI, which looked quite similar to that of the proband. Ophthalmologic examination detected retinal pigmentation, which was otherwise asymptomatic.

Family 9

Proband II.2 from family 9 was a 46-year-old female from Brazil who was referred because of a putative diagnosis of "iron-refractory iron-deficiency anemia" (Figure 1). She had been treated with oral and intravenous iron supplementation for several times since the age of 14 because of microcytic anemia. She also had a clinical history of diabetes mellitus, hypertension, glaucoma, obesity, chronic diarrhea, hypothyroidism, and knee osteoarthritis. Laboratory analysis confirmed microcytic anemia (Hb 9.4 g/dl, MCV 64.5 fl) with elevated ferritin levels (1,060 ng/ml), low serum iron (22 μg/dl), low TSAT (8.2%), and undetectable ceruloplasmin. Brain MRI confirmed iron overload in thalami, dentate nuclei, basal ganglia, and cerebellum. Notably, the patient did not report any overt neurological symptoms.

Family 10

Proband I.1 from family 10 was a 25-year-old female from India who was referred in 2015 for an acute psychotic episode with catatonia, hair loss, and mild dysfunction on formal neurocognitive assessment. She had been previously treated with iron oral supplementation for microcytic anemia. She also had a clinical history of hypothyroidism and amenorrhoea (thought to be due to polycystic ovaries). Laboratory analysis revealed microcytic anemia (Hb 9.2 g/dl, MCV 71.5 fl) with elevated ferritin levels (757 ng/ml), low serum iron (19.5 ug/dl), low TSAT (4%), and undetectable ceruloplasmin. An oral glucose tolerance test was slightly altered. Brain MRI confirmed iron overload in bilateral dentate nuclei thalami, basal ganglia, and choroid plexus. LIC by Ferriscan (a MRI-based approach) was 9.0 mg/g/dw.

Family 11

Proband from Family 12 was a 16-year-old male from Pakistan who was referred in 2015 for concentration/memory lapses. The patient also presented white nails, mild leukopenia, bilateral lattice degeneration of fundi-risk of retinal detachment, and vitamin D depletion. Laboratory analysis revealed elevated ferritin levels (1,065 ng/ml), low serum iron (33.5 ug/dl), low TSAT (8.8%), with normal Hb levels (13.4 g/dl) and undetectable ceruloplasmin. LIC by Ferriscan was 5.3/g/dw.

References

1. Daimon, M., et al., *A novel mutation of the ceruloplasmin gene in a patient with heteroallelic ceruloplasmin gene mutation (HypoCPGM).* Tohoku J Exp Med, 2000. **191**(3): p. 119-25.
2. Haemers, I., et al., *Clinical, molecular, and PET study of a case of aceruloplasminaemia presenting with focal cranial dyskinesia.* J Neurol Neurosurg Psychiatry, 2004. **75**(2): p. 334-7.
3. Hofmann, W.P., et al., *Identification and in silico characterization of a novel compound heterozygosity associated with hereditary aceruloplasminemia.* Scand J Gastroenterol, 2007. **42**(9): p. 1088-94.
4. Kono, S. and H. Miyajima, *Molecular and pathological basis of aceruloplasminemia.* Biol Res, 2006. **39**(1): p. 15-23.
5. Bosio, S., et al., *Anemia and iron overload due to compound heterozygosity for novel ceruloplasmin mutations.* Blood, 2002. **100**(6): p. 2246-8.
6. Kono, S., et al., *Biochemical features of ceruloplasmin gene mutations linked to aceruloplasminemia.* Neuromolecular Med, 2006. **8**(3): p. 361-74.
7. Hellman, N.E., et al., *Biochemical analysis of a missense mutation in aceruloplasminemia.* J Biol Chem, 2002. **277**(2): p. 1375-80.
8. Miyajima, H., Y. Takahashi, and S. Kono, *Aceruloplasminemia, an inherited disorder of iron metabolism.* Biometals, 2003. **16**(1): p. 205-13.
9. Kono, S., *Aceruloplasminemia.* Curr Drug Targets, 2012. **13**(9): p. 1190-9.
10. Okamoto, N., et al., *Hereditary ceruloplasmin deficiency with hemosiderosis.* Hum Genet, 1996. **97**(6): p. 755-8.
11. Hatanaka, Y., et al., *Aceruloplasminemia with juvenile-onset diabetes mellitus caused by exon skipping in the ceruloplasmin gene.* Intern Med, 2003. **42**(7): p. 599-604.
12. Mariani, R., et al., *Iron chelation therapy in aceruloplasminaemia: study of a patient with a novel missense mutation.* Gut, 2004. **53**(5): p. 756-8.
13. Pelucchi, S., et al., *Phenotypic heterogeneity in seven Italian cases of aceruloplasminemia.* Parkinsonism Relat Disord, 2018. **51**: p. 36-42.
14. Shang, H.F., et al., *Novel mutation in the ceruloplasmin gene causing a cognitive and movement disorder with diabetes mellitus.* Mov Disord, 2006. **21**(12): p. 2217-20.
15. Poli, L., et al., *Is aceruloplasminemia treatable? Combining iron chelation and fresh-frozen plasma treatment.* Neurol Sci, 2017. **38**(2): p. 357-360.
16. Perez-Aguilar, F., et al., *Aceruloplasminemia in an asymptomatic patient with a new mutation. Diagnosis and family genetic analysis.* J Hepatol, 2005. **42**(6): p. 947-9.
17. Yazaki, M., et al., *A novel splicing mutation in the ceruloplasmin gene responsible for hereditary ceruloplasmin deficiency with hemosiderosis.* J Neurol Sci, 1998. **156**(1): p. 30-4.
18. Yonekawa, M., et al., *A case of hereditary ceruloplasmin deficiency with iron deposition in the brain associated with chorea, dementia, diabetes mellitus and retinal pigmentation: administration of fresh-frozen human plasma.* Eur Neurol, 1999. **42**(3): p. 157-62.
19. Rusticeanu, M., et al., *Novel ceruloplasmin mutation causing aceruloplasminemia with hepatic iron overload and diabetes without neurological symptoms.* Clin Genet, 2014. **85**(3): p. 300-1.
20. Fasano, A., et al., *Aceruloplasminemia: a novel mutation in a family with marked phenotypic variability.* Mov Disord, 2008. **23**(5): p. 751-5.
21. Miyajima, H., et al., *Familial apoceruloplasmin deficiency associated with blepharospasm and retinal degeneration.* Neurology, 1987. **37**(5): p. 761-7.
22. Harris, Z.L., et al., *Aceruloplasminemia: molecular characterization of this disorder of iron metabolism.* Proc Natl Acad Sci U S A, 1995. **92**(7): p. 2539-43.

23. Meral Gunes, A., et al., *Aceruloplasminemia in a Turkish adolescent with a novel mutation of ceruloplasmin gene: the first diagnosed case from Turkey.* J Pediatr Hematol Oncol, 2014. **36**(7): p. e423-5.
24. Kang, H.G., M. Kim, and S.C. Lee, *Aceruloplasminemia With Positive Ceruloplasm Gene Mutation.* JAMA Ophthalmol, 2016. **134**(7): p. e160816.
25. Pelucchi, S., et al., *Does aceruloplasminemia modulate iron phenotype in thalassemia intermedia?* Blood Cells Mol Dis, 2016. **57**: p. 112-4.
26. Yomono, H., et al., *[An autopsy case of multiple system atrophy with a heteroallelic ceruloplasmin gene mutation].* Rinsho Shinkeigaku, 2003. **43**(7): p. 398-402.
27. Loreal, O., et al., *Aceruloplasminemia: new clinical, pathophysiological and therapeutic insights.* J Hepatol, 2002. **36**(6): p. 851-6.
28. Di Raimondo, D., et al., *Aceruloplasminemia: a case report.* Intern Emerg Med, 2008. **3**(4): p. 395-9.
29. Bethlehem, C., B. van Harten, and M. Hoogendoorn, *Central nervous system involvement in a rare genetic iron overload disorder.* Neth J Med, 2010. **68**(10): p. 316-8.
30. Kerkhof, M. and P. Honkoop, *Never forget aceruloplasminemia in case of highly suggestive Wilson's disease score.* Hepatology, 2014. **59**(4): p. 1645-7.
31. Vroegindeweij, L.H.P., et al., *New insights in the neurological phenotype of aceruloplasminemia in Caucasian patients.* Parkinsonism Relat Disord, 2017. **36**: p. 33-40.
32. Hellman, N.E., et al., *Mechanisms of copper incorporation into human ceruloplasmin.* J Biol Chem, 2002. **277**(48): p. 46632-8.
33. Kohno, S., et al., *Aceruloplasminemia with a novel mutation associated with parkinsonism.* Neurogenetics, 2000. **2**(4): p. 237-8.
34. Melgari, J.M., et al., *Movement disorders and brain iron overload in a new subtype of aceruloplasminemia.* Parkinsonism Relat Disord, 2015. **21**(6): p. 658-60.
35. Lindner, U., et al., *Aceruloplasminaemia: a family with a novel mutation and long-term therapy with deferasirox.* Horm Metab Res, 2015. **47**(4): p. 303-8.
36. Kuhn, J., et al., *Extrapyramidal and cerebellar movement disorder in association with heterozygous ceruloplasmin gene mutation.* J Neurol, 2005. **252**(1): p. 111-3.
37. di Patti, M.C., et al., *Dominant mutants of ceruloplasmin impair the copper loading machinery in aceruloplasminemia.* J Biol Chem, 2009. **284**(7): p. 4545-54.
38. Suzuki, Y., et al., *Effectiveness of oral iron chelator treatment with deferasirox in an aceruloplasminemia patient with a novel ceruloplasmin gene mutation.* Intern Med, 2013. **52**(13): p. 1527-30.
39. Logan, J.I., et al., *Hereditary caeruloplasmin deficiency, dementia and diabetes mellitus.* QJM, 1994. **87**(11): p. 663-70.
40. Miyajima, H., et al., *Estimation of the gene frequency of aceruloplasminemia in Japan.* Neurology, 1999. **53**(3): p. 617-9.
41. Hellman, N.E., et al., *Hepatic iron overload in aceruloplasminaemia.* Gut, 2000. **47**(6): p. 858-60.
42. Finkenstedt, A., et al., *Hepatic but not brain iron is rapidly chelated by deferasirox in aceruloplasminemia due to a novel gene mutation.* J Hepatol, 2010. **53**(6): p. 1101-7.
43. Muroi, R., et al., *Early onset insulin-dependent diabetes mellitus as an initial manifestation of aceruloplasminaemia.* Diabet Med, 2006. **23**(10): p. 1136-9.
44. Nagata, M., et al., *[A case of aceruloplasminemia presenting as cerebellar ataxia with homozygous mutation nt2602 delG].* No To Shinkei, 2004. **56**(10): p. 885-9.
45. Takahashi, Y., et al., *Characterization of a nonsense mutation in the ceruloplasmin gene resulting in diabetes and neurodegenerative disease.* Hum Mol Genet, 1996. **5**(1): p. 81-84.
46. Daimon, M., et al., *A nonsense mutation of the ceruloplasmin gene in hereditary ceruloplasmin deficiency with diabetes mellitus.* Biochem Biophys Res Commun, 1995. **217**(1): p. 89-95.
47. Kawanami, T., et al., *Hereditary caeruloplasmin deficiency: clinicopathological study of a patient.* J Neurol Neurosurg Psychiatry, 1996. **61**(5): p. 506-9.
48. Matsushima, A., et al., *Superficial siderosis associated with aceruloplasminemia. Case report.* J Neurol Sci, 2014. **339**(1-2): p. 231-4.
49. Takeuchi, Y., et al., *A case of aceruloplasminaemia: abnormal serum ceruloplasmin protein without ferroxidase activity.* J Neurol Neurosurg Psychiatry, 2002. **72**(4): p. 543-5.
50. Kono, S., et al., *Hepatic iron overload associated with a decreased serum ceruloplasmin level in a novel clinical type of aceruloplasminemia.* Gastroenterology, 2006. **131**(1): p. 240-5.

51. Yoshida, K., et al., *A mutation in the ceruloplasmin gene is associated with systemic hemosiderosis in humans.* Nat Genet, 1995. **9**(3): p. 267-72.
52. Morita, H., et al., *Hereditary ceruloplasmin deficiency with hemosiderosis: a clinicopathological study of a Japanese family.* Ann Neurol, 1995. **37**(5): p. 646-56.
53. Hida, A., et al., *Aceruloplasminemia in a Japanese woman with a novel mutation of CP gene: clinical presentations and analysis of genetic and molecular pathogenesis.* J Neurol Sci, 2010. **298**(1-2): p. 136-9.
54. Roberti Mdo, R., et al., *Aceruloplasminemia: a rare disease - diagnosis and treatment of two cases.* Rev Bras Hematol Hemoter, 2011. **33**(5): p. 389-92.
55. Servan, J., et al., *[Cerebral hemosiderosis related to hereditary ceruloplasmin deficiency. Clinical familial case study].* Rev Neurol (Paris), 1998. **154**(2): p. 158-62.
56. Dunaief, J.L., et al., *Macular degeneration in a patient with aceruloplasminemia, a disease associated with retinal iron overload.* Ophthalmology, 2005. **112**(6): p. 1062-5.
57. Skidmore, F.M., et al., *Aceruloplasminaemia with progressive atrophy without brain iron overload: treatment with oral chelation.* J Neurol Neurosurg Psychiatry, 2008. **79**(4): p. 467-70.
58. Riboldi, G.M., et al., *Aceruloplasminemia and putaminal cavitation.* Parkinsonism Relat Disord, 2018. **51**: p. 121-123.
59. Watanabe, M., et al., *Aceruloplasminemia with Abnormal Compound Heterozygous Mutations Developed Neurological Dysfunction during Phlebotomy Therapy.* Intern Med, 2018. **57**(18): p. 2713-2718.
60. Stelten, B.M.L., W. van Ommen, and K. Keizer, *Neurodegeneration With Brain Iron Accumulation: A Novel Mutation in the Ceruloplasmin Gene.* JAMA Neurol, 2019. **76**(2): p. 229-230.
